# Association of lipopolysaccharide with new-onset atrial fibrillation in ST-segment elevation myocardial infarction

**DOI:** 10.1016/j.heliyon.2024.e27552

**Published:** 2024-03-11

**Authors:** Honglong Ren, Zhonghua Wang, Yong Li, Jinqi Liu

**Affiliations:** a Department of Gastroenterology, The First People's Hospital of Yuhang District, Hangzhou, 311100, Zhejiang, China; b Department of Cardiology, The First People's Hospital of Yuhang District, Hangzhou, 311100, Zhejiang, China; c Department of Cardiology, Huai'an Second People's Hospital, 223001, Jiangsu, China

## Abstract

**Background:**

Lipopolysaccharide (LPS) is related to various cardiovascular diseases. However, the relationship between LPS and new-onset atrial fibrillation (NOAF) after ST-segment elevation myocardial infarction (STEMI) has yet to be elucidated. This study aimed to evaluate the impact of LPS on NOAF in STEMI patients.

**Methods:**

This was a single-center retrospective observational study including 806 patients diagnosed with STEMI. LPS levels were determined using a commercial ELISA kit. NOAF was characterized by postadmission AF with the absence of any prior history of AF.

**Results:**

A total of 806 participants were enrolled, with 752 individuals in the non-AF group (93.3%) and 54 individuals in the AF group (6.7%). Multivariable analysis showed that LPS (OR = 1.047; 95% CI: 1.029–1.065, P < 0.001) was an independent risk marker for NOAF. The analysis of the ROC demonstrated that LPS had an AUC of 0.717 in predicting NOAF. When LPS was added to the conventional model, the ability of the risk model to discriminate and reclassify NOAF was improved significantly (IDI 0.053, P = 0.001; NRI 0.510, P < 0.001).

**Conclusion:**

Elevated LPS is associated with an increased risk of NOAF in STEMI patients. The integration of LPS can improve the ability to predict NOAF in STEMI patients.

## 1 Introduction

Acute myocardial infarction (AMI) remains one of the leading threats to human health worldwide [1], and atrial fibrillation (AF) is the most prevalent supraventricular arrhythmia in AMI [2]. The incidence of new-onset AF (NOAF) during hospitalization was 6.5–7.9% in patients receiving reperfusion therapy [3,4]. It is noteworthy that all types of AF in AMI can result in a poor prognosis [1]. Unsurprisingly, when both coexist, it poses a significant threat to patients and a therapeutic challenge for physicians. Real-world data have shown that the triple antithrombotic therapy recommended by guidelines currently faces high bleeding risks and low compliance [5]. Therefore, there is a need to investigate more about the risk markers for NOAF after AMI in order to optimize risk stratification and improve prognosis.

Lipopolysaccharide (LPS) is part of the cell wall found in gram-negative bacteria, composed of hydrophobic lipid A and a hydrophilic carbohydrate core [6], and is related to various cardiovascular diseases [7]. The mechanism of AF is complex and includes oxidative stress, inflammation, fibrosis, and ion channel abnormalities [2]. An animal experiment showed that LPS could contribute to the development of atrial arrhythmias by reducing the atrial effective refractory period (ERP) through the suppression of gene expression of the L-type calcium channel [8]. Zhang et al. demonstrated that aging-associated imbalance of the intestinal flora can cause the development of AF, which may be related to the elevated LPS [9]. In a prospective study on AF, LPS has been elucidated to be associated with poorer prognosis [10]. However, the relationship between LPS and NOAF after AMI remains unclear. The purpose of this study was to examine the effect of LPS on NOAF in STEMI patients.

## 2 Methods

### 2.1 Study population

**Fig. 1:**
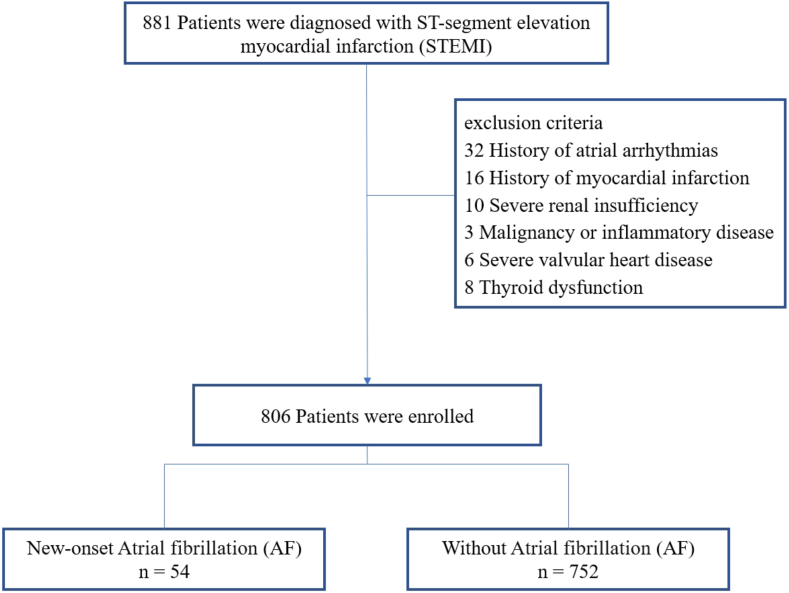
Study flowchart.

In this single-center retrospective observational study, we continuously enrolled patients diagnosed with STEMI [11] at Huai'an Second People's Hospital from January 2019 to May 2023. This study was approved by the ethics committee of Huai'an Second People's Hospital, and signed written consent was waived due to the low risk to the patient. The inclusion criteria were as follows: successful primary percutaneous coronary intervention (pPCI) treatment (TIMI≥2) within 24 h after symptom onset; continuous electrocardiogram monitoring during hospitalization; and serum LPS tested upon admission. Exclusion criteria: under 18 years of age; prior atrial arrhythmias; prior myocardial infarction; significant kidney malfunction (GFR<30 mL/min/1.73 m^2^); malignant or inflammatory diseases; significant valvular heart conditions; and disturbances in thyroid function (hyperthyroidism or subclinical hyperthyroidism, high-sensitivity thyroid stimulating hormone<0.27 mlU/L). Finally, 806 patients were enrolled, and among them, 54 developed new-onset AF (Fig. 1).

### 2.2 Clinical data collection and definition of NOAF

All baseline clinical data for the patients in the study were gathered, including gender, age, body mass index (BMI), smoking, and past medical history. Fasting venous blood was centrifuged and stored at −80 °C until biochemical determination. The levels of serum LPS were assessed through the use of a commercial ELISA kit (Cusabio, Wuhan, China). E. coli-derived LPS standards and blood samples were allowed to incubate for a duration of 2 h at ambient temperature and subsequently dispensed onto microtiter plates that had been pre-coated with antibodies specific to LPS. Following the incubation period, the samples were evaluated at a wavelength of 450 nm. The intra-assay precision was <8%, and the interassay precision was <10%. Values are expressed in pg/mL [10]. Fasting venous blood was collected during hospitalization using an automated analyzer to measure and record high-sensitivity troponin T (hsTnT, ng/L), *N*-terminal pro-B-type natriuretic peptide (NT-proBNP, pg/mL), high-sensitivity c-reactive protein (hs-CRP, mg/L), triglycerides (mmol/L), total cholesterol (mmol/L), high-density leptin cholesterol (mmol/L) and low-density leptin cholesterol (mmol/L). NOAF was characterized by postadmission AF with the absence of any prior history of AF [12]. The infarct-related artery (IRA) was recorded by the coronary angiography results, and all medications given before NOAF onset were recorded. Suspicious rhythms were immediately confirmed as NOAF by 12-lead electrocardiogram monitoring upon admission.

### 2.3 Statistical analysis

Statistical analysis was performed using SPSS 24.0 and R software. Continuous variables following a normal distribution were represented as mean ± standard deviation (SD) and were statistically analyzed using *t*-test; non-normally distributed continuous variables were expressed as median (Q25 Q75) and non-parametric tests (Mann-Whitney *U* test) were used for statistical analysis. All the variables were included in the univariate analysis, and those with a P < 0.05 or clinical significance were further analyzed in the multivariate analysis using forward stepwise regression. Using net reclassification index (NRI) and integrated discrimination index (IDI) to evaluate the additional discriminatory ability of LPS. P < 0.05 was statistically different.

## 3 Results

### 3.1 Baseline characteristics

NOAF occurred in 54 patients (6.7%). The AF group had an older age (71.98 ± 9.42 years vs. 63.11 ± 13.19 years, P < 0.001), a higher proportion of Killip class ≥2 (33.3% vs. 15.0%, P < 0.001), and a lower left ventricular ejection fraction (LVEF) (46.94 ± 9.77% vs. 51.66 ± 6.65%, P < 0.001) than those in the non-AF group. The NT-proBNP [3779.62 (2197.55,6926.35) pg/mL vs. 1979.32 (1050.00,4082.37) pg/mL, P < 0.001] and hs-CRP [76.27 ± 85.69 mg/L vs. 50.87 ± 60.57 mg/L, P = 0.04] were higher in the AF group. Furthermore, the AF group exhibited a significantly elevated level of LPS (65.47 ± 18.16 pg/mL vs. 52.11 ± 16.14 pg/mL, P < 0.001) compared to the non-AF group (Table 1).

**Table 1:** Baseline characteristics of the study population.

	Non-AF (n = 752)	AF (n = 54)	*P*
Age, years	63.11 ± 13.19	71.98 ± 9.42	**<0.001**
Male, n (%)	545(72.5%)	36(66.7%)	0.358
BMI, kg/m^2^	24.55 ± 3.44	25.33 ± 4.91	0.120
Heart rate, bpm	79.30 ± 14.00	82.78 ± 14.34	0.079
SBP, mmHg	126.84 ± 21.05	122.69 ± 27.87	0.171
DBP, mmHg	78.66 ± 13.63	76.85 ± 15.26	0.351
LVEF, %	51.66 ± 6.65	46.94 ± 9.77	**<0.001**
hsTnT, ng/L	2884.0(1244.3,5657.8)	3896.0(1447.0,6686.0)	0.080
NT-proBNP, pg/mL	1979.3(1050.0,4082.4)	3779.6(2197.6,6926.4)	**<0.001**
hs-CRP, mg/L	50.87 ± 60.57	76.27 ± 85.69	**0.004**
Triglycerides, mmol/L	4.43 ± 1.06	4.22 ± 1.13	0.184
Total cholesterol, mmol/L	1.44 ± 0.89	1.54 ± 1.28	0.062
Killip class ≥2, n (%)	113(15.0%)	18(33.3%)	**<0.001**
HDL, mmol/L	1.06 ± 0.28	1.01 ± 0.19	0.198
LDL, mmol/L	2.77 ± 0.90	2.54 ± 0.85	0.069
Hypertension, n (%)	316(42.0%)	26(48.1%)	0.379
Diabetes, n (%)	183(24.3%)	14(25.9%)	0.793
Stroke, n (%)	88(11.7%)	4(7.4%)	0.338
Smoking, n (%)	311(41.4%)	18(33.3%)	0.247
Medication			
Aspirin, n (%)	689(91.6%)	53(98.1%)	0.087
Beta-blocker, n (%)	604(80.3%)	46(85.2%)	0.382
ACEI/ARB/ARNI, n (%)	390(51.9%)	30(55.6%)	0.600
P_2_Y_12_ inhibitors, n (%)	717(95.3%)	54(100.0%)	0.105
Statin, n (%)	705(93.8%)	54(100.0%)	0.058
Infarct-related artery			
LAD, n (%)	389(51.7%)	21(38.9%)	0.068
LCX, n (%)	87(11.6%)	9(16.7%)	0.264
RCA, n (%)	253(33.6%)	23(42.6%)	0.181
LPS, pg/mL	52.11 ± 16.14	65.47 ± 18.16	**<0.001**

BMI, Body mass index; SBP, systolic blood pressure; DBP, Diastolic blood pressure; hs-TnT, high-sensitivity troponin T; NT-proBNP, N terminal pro-B-type natriuretic peptide; hs-CRP, high-sensitivity C-reactive protein; LDL cholesterol, low density leptin cholesterol; HDL cholesterol, high density leptin cholesterol; ACEI, angiotensin-converting-enzyme inhibitor; ARB, angiotensin II receptor blocker; ARNI, angiotesin receptor-neprilysin inhibitor; LVEF, left ventricular ejection fraction; LAD, left anterior descending; LCX, left circumflex artery; RCA, right coronary artery; LPS, lipopolysaccharide.

### 3.2 Univariate and multivariate logistic regression analysis

Univariate analysis showed that age, hs-CRP, NT-proBNP, LVEF, Killip class ≥2, left anterior descending (LAD), and LPS were associated with NOAF (P < 0.05). Multivariable analysis indicated that age, LVEF, LAD, and LPS (OR = 1.047; 95% CI: 1.029–1.065, P < 0.001) were independent risk markers for NOAF (Table 2).

**Table 2:** Univariate and multivariate logistic regression analysis.

	Univariate analysis		Multivariate analysis	
	*OR (95%CI)*	*P*	*OR (95%CI)*	*P*
Age, years	1.065(1.037–1.094)	**<0.001**	1.064(1.035–1.093)	**<0.001**
Male, n (%)	1.316(0.731–2.370)	0.359		
BMI, kg/m^2^	1.061(0.985–1.144)	0.120		
Heart rate, bpm	1.018(0.998–1.038)	**0.080**		
SBP, mmHg	0.991(0.979–1.004)	0.170		
DBP, mmHg	0.990(0.970–1.011)	0.351		
LVEF, %	0.921(0.890–0.953)	**<0.001**	0.923(0.889–0.958)	**<0.001**
hsTnT, ng/L	1.000(1.000–1.000)	0.129		
NT-proBNP, pg/mL	1.000(1.000–1.000)	**0.007**		
hs-CRP, mg/L	1.005(1.002–1.009)	**0.005**		
Triglycerides, mmol/L	0.829(0.628–1.093)	0.183		
Total cholesterol, mmol/L	1.102(0.844–1.439)	0.476		
Killip class ≥2, n (%)	2.827(1.552–5.152)	**0.001**		
HDL, mmol/L	0.435(0.125–1.509)	0.190		
LDL, mmol/L	0.730(0.520–1.023)	**0.068**		
Hypertension, n (%)	1.281(0.737–2.228)	0.380		
Diabetes, n (%)	1.088(0.579–2.045)	0.793		
Stroke, n (%)	0.604(0.213–1.712)	0.343		
Smoking, n (%)	0.709(0.395–1.272)	0.249		
Aspirin, n (%)	4.846(0.659–35.634)	0.121		
Beta-blocker, n (%)	1.409(0.651–3.049)	0.384		
ACEI/ARB/ARNI, n (%)	1.160(0.666–2.022)	0.600		
P_2_Y_12_ inhibitors, n (%)	121667665.7(0.000-)	0.998		
Statin, n (%)	123738561.7(0.000-)	0.997		
LAD, n (%)	0.594(0.337–1.045)	**0.071**	0.456(0.245–0.849)	**0.013**
LCX, n (%)	1.529(0.722–3.236)	0.267		
RCA, n (%)	1.463(0.836–2.562)	0.183		
LPS, pg/mL	1.044(1.028–1.061)	**<0.001**	1.047(1.029–1.065)	**<0.001**

BMI, Body mass index; SBP, systolic blood pressure; DBP, Diastolic blood pressure; hs-TnT, high-sensitivity troponin T; NT-proBNP, N terminal pro-B-type natriuretic peptide; hs-CRP, high-sensitivity C-reactive protein; LDL cholesterol, low density leptin cholesterol; HDL cholesterol, high density leptin cholesterol; ACEI, angiotensin-converting-enzyme inhibitor; ARB, angiotensin II receptor blocker; ARNI, angiotesin receptor-neprilysin inhibitor; LVEF, left ventricular ejection fraction; LAD, left anterior descending; LCX, left circumflex artery; RCA, right coronary artery; LPS, lipopolysaccharide.

### 3.3 Receiver operating characteristic

**Fig. 2:**
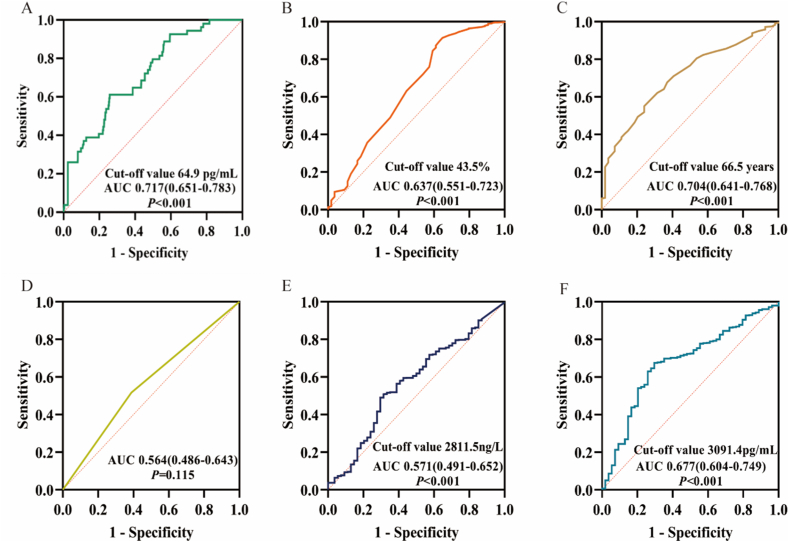
ROC (receiver operating characteristic) predicting the new-onset atrial fibrillation (NOAF) in ST-segment elevation myocardial infarction patients undergoing primary percutaneous coronary intervention. A, ROC about Lipopolysaccharide (LPS); B, ROC about left ventricular ejection fraction (LVEF); C, ROC about age; D, ROC about left anterior descending (LAD); E, ROC about high-sensitivity troponin T (hsTnT); F, ROC about *N*-terminal pro-B-type natriuretic peptide (NT-proBNP).

ROC curve analysis showed that LVEF, LAD, hsTnT, NT-proBNP, age and LPS (AUC 0.717, 95% CI 0.651–0.783) had some predictive value for NOAF. Of these, LPS and age showed good predictive value for NOAF, and the cutoff value for LPS was 64.9 pg/mL (Fig. 2A–F).

### 3.4 Incremental value of LPS to NOAF

A conventional model was constructed using independent risk markers identified by multivariable analysis, which included age, LVEF and LAD. When LPS was added to the conventional model, the ability of the risk model to discriminate and reclassify NOAF was improved (IDI 0.053, P = 0.001; NRI 0.510, P < 0.001) (Table 3).

**Table 3:** Discrimination accuracy and reclassification of risk markers of NOAF.

	NRI		IDI	
	Estimate (95% CI)	P	Estimate (95% CI)	P
Conventional model	Reference	–	Reference	–
Conventional model + LPS, continuous	0.510 (0.241–0.778)	<0.001	0.053 (0.021–0.085)	0.001

Conventional model included Age, left ventricular ejection fraction, and left anterior descending.

LPS, lipopolysaccharide; CI, confidence interval; IDI, integrated discrimination index; NRI, net reclassification improvement.

## 4 Discussion

The key results of this study were outlined below. First, higher LPS was still associated with NOAF after adjusting for potential confounders. Second, LPS was able to predict the occurrence of NOAF satisfactorily. Besides, LPS could significantly improve the ability of the risk model to discriminate and reclassify NOAF.

STEMI remains one of the primary cause of mortality among residents worldwide, and AF is often linked to higher risks of stroke and heart failure [1,2]. Therefore, it is not surprising that the combination of the two has attracted widespread attention and vigilance. Similar to some other studies, the occurrence of NOAF during hospitalization was 6.7% in this study. In the RISK-PCI study, the occurrence of NOAF in acute myocardial infarction patients receiving pPCI treatment was 6.2% [13]. In the GUSTO III trial, NOAF developed in 6.5% of patients during hospitalization [4]. Age as a risk factor for AF seems to be undisputed, and this is also true in the acute myocardial infarction population [3,14,15]. The mutual influence between AF and LVEF has also been widely demonstrated in acute myocardial infarction patients [3,13]. Similarly, we found that older age and lower LVEF were linked to NOAF during hospitalization in STEMI patients who underwent pPCI treatment. In addition, we found that when the IRA was LAD, it appeared to be a protective factor. A previous study demonstrated that atrial ischemia was one of the mechanisms leading to AF after myocardial infarction [16]. A study on coronary arteries showed that AMI patients who had atrial tachycardia often had atrial infarction. In 50–60% of cases, the atrial blood supply came from the sinoatrial branch of the right coronary artery, while in 40–50% of cases, it came from the left circumflex artery [17]. The risk of NOAF after myocardial infarction was higher when the diseased coronary artery involves the blood supply to the atria [18,19]. In contrast, although the LAD provides a considerable amount of ventricular muscle, it is not of great importance to the atrium [20]. Therefore, when the IRA is LAD, it may be a protective factor compared to other patients with IRA in the LCX or RCA.

The gut microbiota and their metabolites were associated with inflammation and promote the occurrence and development of AF [21,22]. In a prospective single-center study, it was found that patients with AF had higher LPS levels compared to those in sinus rhythm, and in AF patients, LPS might cause MACE by increasing platelet activation [10]. Wang et al. discovered that the gut microbiome significantly impacted the prognosis after AF ablation, and LPS was a predictive factor for AF recurrence during a one-year follow-up [23]. Consistent with these study results, our findings indicate that higher LPS was significantly associated with NOAF during hospitalization (OR = 1.047, 95% CI: 1.029–1.065, P < 0.001). This may be related to the following reasons. A previous study showed that in different cardiac metabolic diseases, LPS could increase the reactive oxygen species by acting on multiple pathways [24]. Menichelli et al. confirmed in the ATHERO-AF study that circulating LPS could cause oxidative stress [25]. Toll-like receptor (TLR) 4 is a key genetic hub for the occurrence of AF and is notably increased in the atria when AF occurs [26,27]. The LPS-TLR4 interaction is an important mechanism leading to systemic inflammation. The LPS-treated rabbit pericardium had higher expression of proinflammatory cytokines, and LPS promoted the occurrence of atrial arrhythmias through its interaction with TLR4 [28,29]. In an animal study, LPS-associated inflammation was found to lead to atrial conduction heterogeneity, which increased the likelihood of AF recurrence [30]. These studies indicate that oxidative stress and the inflammatory response may be important mechanisms for LPS-induced atrial fibrillation. In addition, LPS is also related to the remodeling of atria. Okazaki et al. injected LPS intraperitoneally into 10-week-old Sprague‒Dawley rats and found that the mRNA levels of L-type calcium channel genes were significantly decreased at 6 and 12 h after LPS injection, which significantly shortened the atrial ERP [8]. Previous studies found that LPS could directly change the ionic current function of myocardial cells, contributing to an arrhythmia axis [31,32]. In an animal study, dysbiosis of the intestinal flora was shown to partially induce atrial fibrillation by increasing circulating LPS levels, thereby promoting atrial fibrosis [9]. Wang et al. discovered a positive association between circulating LPS levels and serum TGF-β1 levels, the latter of which was known to be a significant factor in inducing myocardial fibrosis [23,33]. Finally, in a study on AMI, Zhou et al. showed that increased levels of LPS were linked to dysfunction in the left ventricle and could predicted adverse cardiovascular events [34], which may also be one of the potential reasons for the association between LPS and NOAF. In this study, we also found that LPS could predict the occurrence of NOAF satisfactorily (AUC 0.717). When LPS was added to the conventional model, the ability to discriminate and reclassify NOAF was improved significantly (IDI 0.053, P = 0.001; NRI 0.510, P < 0.001). This may help optimize the risk stratification of NOAF in STEMI patients after pPCI treatment and identify such high-risk patients.

### 4.1 STUDY LIMITATIONS and STRENGTHS

This research has several limitations. First, it was a retrospective study conducted at a single center, and the generalization of the conclusions may require more multicenter randomized controlled trials (RCTs). Second, we analyzed NOAF events during hospitalization in this study, while long-term AF after discharge may have greater clinical value for patients, and the value of LPS for this group of patients may need more studies to clarify. Third, the participants analyzed in this study consisted of patients with STEMI who received pPCI intervention, so the results of this study may not be applicable to all populations.

This study also has several strengths. First, to our knowledge, this investigation was the inaugural assessment of the effects of LPS on NOAF in STEMI patients. We found that LPS was associated with NOAF in STEMI patients and can improve the original NOAF risk model, which may help in the early identification and risk stratification of this high-risk group of patients. Second, all patients in this study received continuous cardiovascular blood pressure pulse oximetry monitoring during their hospitalization, which may have reduced the omission of AF events to some extent.

## 5 Conclusion

Elevated LPS is associated with an increased risk of NOAF in STEMI patients. The integration of LPS can improve the ability to predict NOAF in STEMI patients.

## Ethics statement

The study was reviewed by the Ethics Committee of the Huai'an Second People's Hospital (APPROVAL NUMBER: HEYLL202317).

## Data availability statement

The data of this study are available from the corresponding author by reasonable request.

## Funding statement

Hangzhou Science and Technology Plan Steering Project (20201231Y151).

## CRediT authorship contribution statement

**Honglong Ren:** Writing – original draft, Methodology, Data curation, Conceptualization. **Zhonghua Wang:** Methodology, Investigation, Data curation. **Yong Li:** Supervision, Resources, Methodology, Funding acquisition. **Jinqi Liu:** Writing – review & editing, Supervision, Project administration, Methodology, Conceptualization.

## Declaration of competing interest

The authors declare that they have no known competing financial interests or personal relationships that could have appeared toinfluence the work reported in this paper.
